# Transcription start site‐level expression of thyroid transcription factor 1 isoforms in lung adenocarcinoma and its clinicopathological significance

**DOI:** 10.1002/cjp2.213

**Published:** 2021-05-20

**Authors:** Kei Sano, Takuo Hayashi, Yoshiyuki Suehara, Masaki Hosoya, Kazuya Takamochi, Shinji Kohsaka, Satsuki Kishikawa, Monami Kishi, Satomi Saito, Fumiyuki Takahashi, Kazuo Kaneko, Kenji Suzuki, Takashi Yao, Muneaki Ishijima, Tsuyoshi Saito

**Affiliations:** ^1^ Department of Human Pathology Juntendo University Graduate School of Medicine Tokyo Japan; ^2^ Department of Medicine for Orthopaedics and Motor Organ Juntendo University Graduate School of Medicine Tokyo Japan; ^3^ Department of Medical Oncology Juntendo University Graduate School of Medicine Tokyo Japan; ^4^ Department of General Thoracic Surgery Juntendo University Graduate School of Medicine Tokyo Japan; ^5^ Division of Cellular Signaling National Cancer Center Research Institute Tokyo Japan; ^6^ Department of Respiratory Medicine Juntendo University Graduate School of Medicine Tokyo Japan

**Keywords:** lung adenocarcinoma, TTF‐1, NKX2‐1, promoter, 5′‐UTR, TSS, isoforms

## Abstract

There are multiple transcription start sites (TSSs) in agreement with multiple transcript variants encoding different isoforms of NKX2‐1/TTF‐1 (thyroid transcription factor 1); however, the clinicopathological significance of each transcript isoform of *NKX2‐1/TTF‐1* in lung adenocarcinoma (LAD) is unknown. Herein, TSS‐level expression of *NKX2‐1/TTF‐1* isoforms was evaluated in 71 LADs using bioinformatic analysis of cap analysis of gene expression (CAGE)‐sequencing data, which provides genome‐wide expression levels of the 5′‐untranslated regions and the TSSs of different isoforms. Results of CAGE were further validated in 664 LADs using *in situ* hybridisation. Fourteen of 17 TSSs in *NKX2‐1/TTF‐1* (80% of known TSSs in FANTOM5, an atlas of mammalian promoters) were identified in LADs, including TSSs 1–13 and 15; four isoforms of *NKX2‐1/TTF‐1* transcripts (*NKX2‐1_001*, *NKX2‐1_002*, *NKX2‐1_004*, and *NKX2‐1_005*) were expressed in LADs, although *NKX2‐1_005* did not contain a homeodomain. Among those, six TSSs regulated *NKX2‐1_004* and *NKX2‐1_005*, both of which contain exon 1. LADs with low expression of isoforms from TSS region 11 regulating exon 1 were significantly associated with poor prognosis in the CAGE data set. In the validation set, 62 tumours (9.3%) showed no expression of *NKX2‐1/TTF‐1* exon 1; such tumours were significantly associated with older age, *EGFR* wild‐type tumours, and poor prognosis. In contrast, 94 tumours, including 22 of 30 pulmonary invasive mucinous adenocarcinomas (IMAs) exhibited exon 1 expression without immunohistochemical TTF‐1 protein expression. Furthermore, IMAs commonly exhibited higher exon 1 expression relative to that of exon 4/5, which contained a homeodomain in comparison with *EGFR‐*mutated LADs. These transcriptome and clinicopathological results reveal that LAD use at least 80% of *NKX2‐1* TSSs and expression of the *NKX2‐1/TTF‐1* transcript isoform without exon 1 (*NKX2‐1_004* and *NKX2‐1_005*) defines a distinct subset of LAD characterised by aggressive behaviour in elder patients. Moreover, usage of alternative TSSs regions regulating *NKX2‐1_005* may occur in subsets of LADs.

## Introduction

Thyroid transcription factor 1 (TTF‐1), also known as NKX2‐1, is a member of the highly conserved homeodomain‐containing transcription factor family, which activates the expression of selected genes in the lung and thyroid, as well as a restricted part of the brain, and is essential for the development and differentiation of these organs [1, 2, 3]. The cellular mechanisms regulating lung homeostasis are not completely understood; however, different epithelial regions and compartments in the lung are known to be maintained by distinct resident stem cell populations [4]. Notably, a series of peripheral lung cells defined as the terminal respiratory unit (TRU), in which gas exchange occurs, is under the control of *NKX2‐1/TTF‐1* [5]. Furthermore, approximately 70% of lung adenocarcinomas (LADs) express TTF‐1 independent of disease stage and retain features of the TRU to a certain extent [6], strongly suggesting that *NKX2‐1/TTF‐1* is a potential lineage survival oncogene in lung cancer [7]. Currently, TTF‐1 is widely used as the most specific marker for LAD diagnosis in routine pathological examination [8], which leads to the detection of actionable alterations, such as *EGFR*, *KRAS*, and *BRAF* mutations; gene fusions involving anaplastic lymphoma kinase (*ALK*); rearranged during transfection (*RET*); and proto‐oncogene tyrosine‐protein kinase ROS (*ROS1*) or tyrosine‐protein kinase Met (*MET*) exon 14 skipping [9, 10]. Despite the discovery of these oncogene mutations, at least 12% of patients with LAD do not possess any of these genetic alterations [10, 11], suggesting that other molecular changes likely contribute to lung cancer development.

Epigenomic features do not affect the DNA sequence, but may affect the transcriptional output of genes in a cell‐type specific manner by altering the activity of regulatory elements, including promoters, which are located proximal to the transcription start site (TSS) of genes [12]. Alternative splicing is the process by which a single gene may produce many different transcripts that may show a wide range of activities, and is responsible for much of the diversity of the human proteome [12]. TSS determination of *NKX2‐1/TTF‐1* transcription shows multiple TSSs, in agreement with multiple transcript variants encoding different isoforms [5]. In humans, two complementary DNAs (cDNAs) were initially identified that translate into the 42‐kDa ‘major isoform’ and the 44‐kDa ‘minor protein isoform’. These isoforms were differentially expressed during mouse fetal lung development, with the onset of accumulation of the longer transcript occurring at a later stage than that of the shorter transcript [13, 14]. These two transcripts have differences in their capacity to activate the surfactant protein‐C promoter, which is a pulmonary differentiation‐specific gene, indicating functional differences [13]. Furthermore, *NKX2‐1/TTF‐1* shows different functions depending on cell conditions, being considered a double‐sword gene with lineage‐dependent tumour cell survival and tumour suppression activities depending on the context [7]. This suggests that each isoform has differential functions in lung carcinogenesis. However, the clinicopathological significance of each promoter and the concordant isoform in LAD remains largely unknown.

In recent studies, an atlas of human cellular states based on regulatory element activities across the genome, such as promoters [15] and enhancers [16], has been built by monitoring transcription initiation activities with cap analysis of gene expression (CAGE) [17]. The method determines 5′‐end sequences of messenger RNA (mRNA) using next‐generation sequencing, where cDNAs are synthesised from extracted RNA, and cDNAs corresponding to 5′‐ends of RNA are selected using the cap‐trapper method [18] and sequenced. Obtained reads are aligned with genome sequences and their 5′‐ends indicate frequencies of TSSs at single‐base resolution [19]. Herein, the ability of this technology to elucidate the role of each TSS and transcript isoform of *NKX2‐1/TTF‐1* in LAD was examined, with special emphasis on its prognostic impact. The clinicopathological significance of *NKX2‐1/TTF‐1* exon 1 expression in a large cohort of Japanese patients was further evaluated using RNAscope, a novel *in situ* hybridisation assay. We used the *NKX2‐1* probe that was designed to target exon 1 in *NKX2‐1_004* (ENST00000518149.5_4). This study expands the understanding of the role of *NKX2‐1/TTF‐1* in LAD.

## Materials and methods

### Study population

The archives of the Department of Human Pathology, Juntendo University School of Medicine, were screened for all patients who had undergone a complete resection of primary LAD from February 2010 to July 2016. Clinicopathological data were obtained, including age, gender, smoking status, tumour size, lymphovascular invasion, lymph node and distant metastases, resection type, adjuvant therapy, and mutation status of *EGFR* and *KRAS*. The archives contained data for 1,124 patients with LAD. Of the 1,124 LAD samples of the cohort, 71 cases were assigned to the discovery set used to perform CAGE assay [20], while full‐length RNA sequencing (RNA‐seq) was also performed in seven cases [9]. Among the remaining 1,053 cases, adenocarcinoma *in situ*, minimally invasive adenocarcinoma, and lepidic adenocarcinoma were excluded to clarify the prognostic impact of *NKX2‐1* exon 1 expression. Invasive LADs with intermediate‐ to high‐grade clinical aggressiveness including acinar, papillary, solid, micropapillary, or other invasive adenocarcinomas of a special type [21] were assigned to the validation set. Follow‐up was conducted for all patients via regular physical and blood examination, with mandatory X‐ray, computed tomography, or magnetic resonance imaging. Informed consent was obtained from all involved patients. The study design was ethically approved by the institutional review board of Juntendo University (Approval No. 2020096).

### Bioinformatics analysis of the CAGE data set

CAGE data were obtained from a previous study [20]. In brief, the CAGE reads were aligned to the reference genome (hg19) with a high mapping quality of ≥20. The aligned CAGE reads were counted in each region of the FANTOM5 robust peaks [15], a reference set of TSS regions, as raw signals for promoter activities. Expression levels of individual TSSs were quantified as counts per million (CPM). Inactive TSS regions, with CPM ≤ 1 in more than 77% of samples, were filtered out [22]. Associations among the TSS regions were assessed by Spearman's rank correlation. The distances between the samples in the *NKX2‐1* TSS regions were calculated as Euclidean distances for CPM, and the average linkage clustering was performed using R (version 3.6.3, https://www.r-project.org/). Based on expression levels, survival analyses of individual TSS regions were performed using the survival package in R (https://cran.r-project.org/web/packages/survival/).

### Histological and immunohistochemical analyses

All tissues were fixed in 10% formalin‐fixed paraffin‐embedded (FFPE) after routine processing. Haematoxylin and eosin (H&E)‐stained slides and Elastica van Gieson‐stained slides were available for all patient samples. All tumours measuring 3 cm or less in diameter were submitted in their entirety, and larger tumours were sampled extensively. Pathological diagnoses were based on the 2015 World Health Organization classification [23]. For immunohistochemical analyses of TTF‐1 (clone 8G7G3/1; DAKO, Glostrup, Denmark), tumours were assembled into tissue microarrays (TMAs), using 1.5–2.0 mm cores sampled from one or two different representative areas of each FFPE tissue block (Pathology Institute Corp., Toyama, Japan), as previously described [24]. TTF‐1 was considered positive if 1% or greater of tumour cells were stained.

### RNAscope assay and image analysis

*In situ* detection of *NKX2‐1* transcript was performed with a RNAscope Assay using the RNAscope Duplex Reagent Kit (#322430; Advanced Cell Diagnostics Inc., Newark, CA, USA), according to the manufacturer's instructions. The *NKX2‐1* probe was designed to target exon 1 in *NKX2‐1_004* (ENST00000518149.5_4) (Advanced Cell Diagnostics Inc.) (see supplementary material, Table S1). For the RNAscope assay, TMA slides from FFPE tissue blocks were used. RNAscope and immunohistochemistry for TTF‐1 were performed on serial sections. The 664 cases of the validation cohort and 33 cases of the discovery cohort were submitted to RNAscope assay. To ensure result interpretability, a positive (#313901, RNAscope Positive Control Probe‐Hs‐PPIB; Advanced Cell Diagnostics Inc.) and a negative control probe (#310043, RNAscope Negative Control Probe‐Hs‐DapB; Advanced Cell Diagnostics Inc.) were used. After staining, TMA slides were scanned using the Nuance Multispectral Imaging System (version 3.0.2; Perkin Elmer Inc., Waltham, MA, USA). inForm Advanced Image Analysis Software (version 2.4.0; Perkin Elmer Inc.) was used for quantitative image analysis. Four random areas (0.09048 mm^2^ each) in each sample were analysed at ×400 total magnification. The data are expressed as optical density (average signal levels per area). An optical density of ≥66.56 was considered positive for expression based on the optical density distribution data in the 664 samples examined in this study (see supplementary material, Figure S1).

### Quantitative polymerase chain reaction

Quantitative polymerase chain reaction (qPCR) was performed on 20 cases of invasive mucinous adenocarcinoma (IMA) of the lung whose genetic alterations were previously described [25], as well as three *EGFR*‐mutated LAD cases that were immunohistochemically positive for TTF‐1, consisting of two cases of papillary adenocarcinoma and one case of acinar adenocarcinoma. RNA was extracted from FFPE tissue using the RNeasy FFPE Kit (Qiagen, Hilden, Germany). qPCR was performed using inventoried Taqman assays (Applied Biosystems, Carlsbad, CA, USA) corresponding to exon 1 in the *NKX2‐1_004* isoform (forward: 5′‐GCCATTTACGCCACCACTTTAA‐3′; reverse: 5′‐GCAGCTCAGCCATGCAAA‐3′; probe: AAGATATTTGGTTATTCCCG), *TTF‐1* exon 4/5 (Assay ID: Hs00968940_m1; Thermo Fisher Scientific, Waltham, CA, USA), myosin‐binding protein H (*MYBPH*) (Assay ID: Hs00192226_m1; Thermo Fisher Scientific), and glyceraldehyde 3‐phosphate dehydrogenase (*GAPDH*) (Assay ID: Hs02786624_g1; Thermo Fisher Scientific). All PCRs were performed with a TaqMan Fast Advanced Master Mix (Applied Biosystems) on an Applied Biosystems Step One Plus Real‐Time PCR System in accordance with the standard protocols. The amount of each target gene relative to the *GAPDH* housekeeping gene was determined using the comparative threshold cycle (Ct) method. Data are the mean of values from three separate experiments.

### Cell lines and cell culture

*EGFR*‐mutated cell lines (H3255, 11–18, H4006, HCC827, PC9, and H1650) and *KRAS*‐mutated cell lines (HCC44, H23, H2030, and A549) were purchased from the American Type Culture Collection (Manassas, VA, USA). All cell lines were cultured in RPMI‐1640 supplemented with 10% FBS and 1% penicillin–streptomycin in an atmosphere of 5% CO_2_ at 37 °C. All cell lines were routinely tested for Mycoplasma and were found to be negative.

### Western blot assays

Protein samples were separated by SDS‐PAGE and subsequently blotted onto a polyvinylidene fluoride membrane. An iBind Western Device (Life Technologies Corporation, Carlsbad, CA, USA) was used for the antigen–antibody reaction. The membrane was incubated with antibodies against TTF‐1 (#sc‐53 136; Santa Cruz, Dallas, TX, USA) and GAPDH (#sc‐32 233; Santa Cruz). Bound antibodies were detected with horseradish peroxidase‐conjugated secondary mouse antibody (GE Healthcare Biosciences, Little Chalfont, UK), and images were taken using the Amersham Imager 680 (GE Healthcare Biosciences).

### Statistical analysis

Categorical variables were analysed using a Fisher's exact or chi‐square test. To determine prognosis, Kaplan–Meier survival analysis was performed. The date of surgical resection was set as the starting point and the date of death, date of recurrence, or last date of follow‐up was used as the end point. Statistical analyses were performed using GraphPad Prism® software version 7.0a (GraphPad, San Diego, CA, USA). *P* value of <0.05 was considered significantly different.

## Results

### Clinicopathological characteristics in the study cohort

Clinicopathological characteristics of 71 and 664 patients with LADs examined in the discovery and validation data sets, respectively, are shown in Table 1. The median age of the 71 cases in the discovery data set was 66.7 years, 29 (40.8%) of which were female and 37 (52.9%) were never or light smokers (the smoking index was ≤400); the median age of the 664 cases in the validation data set was 67.8 years, 314 (47.2%) of which were female and 367 (55.4%) were never or light smokers. Overall, comparison of the discovery and validation data sets revealed no significance differences in clinicopathological features, such as age, gender, smoking status, and the pathological stage at presentation. As expected, histological subtypes were significantly different among the two groups as the validation data set consisted of invasive LADs, including acinar, papillary, solid, micropapillary, and other invasive adenocarcinomas of a special type.

**Table 1:** Clinicopathological characteristics of patients with LAD.

Characteristics	Discovery set (*n* = 71)	Validation set (*n* = 664)	Correlation (*p*)
Median age (range)	66.72 (34–86)	67.78 (28–89)	0.3387
Gender			0.3009
Female	29	314	
Male	42	350	
Smoking index			0.9065
≤100	31	297	
101–400	6	70	
≥401	33	295	
Unknown	0	2	
Size (mm)			0.2975
≤20	33	236	
21–30	19	200	
31–50	12	158	
≥51	7	70	
Nodal status			0.4945
N0	53	470	
N1/N2/N3	18	194	
TNM stage			0.6949
0–I	45	405	
II–IV	26	259	
Histological subtype			<0.0001
Adenocarcinoma *in situ*	3	0	
Minimally invasive adenocarcinoma	6	0	
Lepidic adenocarcinoma	21	0	
Acinar adenocarcinoma	22	305	
Papillary adenocarcinoma	3	206	
Solid adenocarcinoma	10	111	
Micropapillary adenocarcinoma	0	7	
IMA	5	30	
Enteric adenocarcinoma	0	3	
Fetal adenocarcinoma	0	2	
Lymphovascular invasion			0.6737
Absent	39	382	
Present	32	282	
Pleural invasion			0.2435
Absent	50	418	
Present	21	246	
Micropapillary pattern			0.1383
Absent	66	572	
Present	5	92	
Cribriform pattern			0.1360
Absent	68	598	
Present	3	66	
Clear cell features			0.0869
Absent	65	556	
Present	6	108	
Signet ring cell features			>0.9999
Absent	71	655	
Present	0	9	
TTF‐1 immunoreactivity			>0.9999
Absent	12	114	
Present	59	550	
*EGFR* mutation			0.1605
Absent	36	394	
Present	35	270	
*KRAS* mutation			0.9499
Absent	60	563	
Present	11	101	

### CAGE assay demonstrated TSS‐level expression of *NKX2‐1/TTF‐1* in LAD

**Figure 1:**
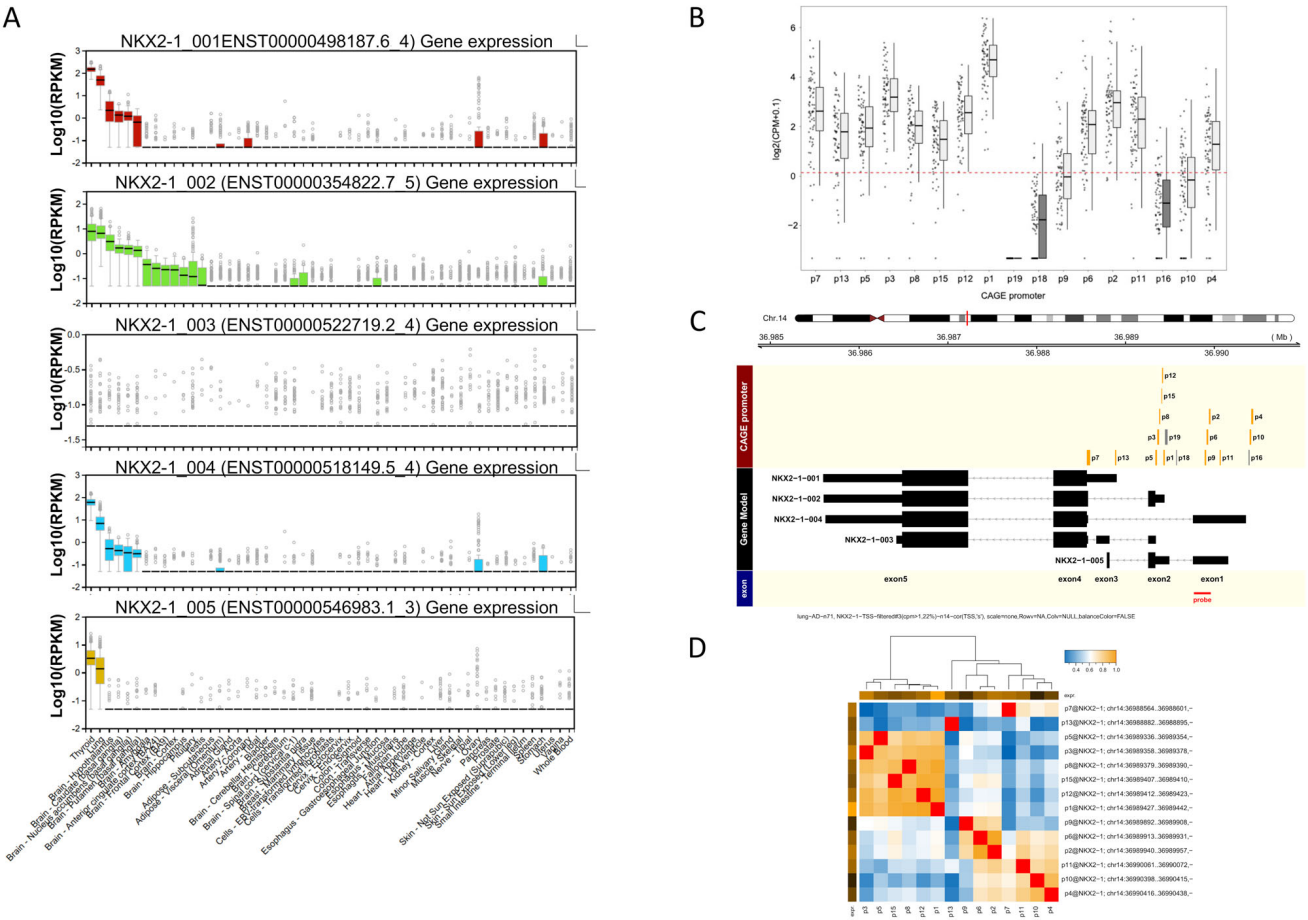
The structure of human TTF‐1 (*NKX2‐1/TTF‐1)* and its transcript isoforms. (A) Genotype‐Tissue Expression (GTEx) analysis release V6p (dpGap Accession phs000424.v6.p1) shows four of five transcript isoforms that are highly expressed in the normal lung and thyroid tissues. (B) Box plots of the TSS regions of *NKX2‐1/TTF‐1* in the discovery set of 71 LADs. Expression is quantified as CPM. Each box indicates the mean and upper and lower quartiles, whereas the bar indicates the range. The threshold (log2[1.1]) is indicated by the red line. Expression of 14 TSSs (82%) is detected, while expression of TSSs 16, 18, and 19 is not detected. (C) The structure of human *NKX2‐1/TTF‐1* and its TSSs. Yellow and grey boxes indicate TSS regions. The wide and narrow black boxes indicate coding exons, and 5′‐ or 3′‐UTRs, respectively. The red bar indicates the targeted region of the probe for RNAscope. (D) Correlation analysis of the expression level of each TSS using Spearman's rank correlation in the discovery set of 71 LADs shows that the TSSs regulating the same exon are correlated with each other.

Quantitative TSS‐level expression profiles were obtained from 71 LADs in the discovery data set in which the CAGE assay was performed as previously described [20]. Initially, genome databases were explored, such as Ensemble, UCSC Genome Browser, and NCBI Human Genome Resources, to obtain the location and gene structure of human *NKX2‐1/TTF‐1*. Human *NKX2‐1/TTF‐1* is located on chromosome 14 and produces five transcripts (ENST00000498187.6_4, *NKX2‐1_001*; ENST00000354822.7_5, *NKX2‐1_002*; ENST00000522719.2_4, *NKX2‐1_003*; ENST00000518149.5_4, *NKX2‐1_004*; and ENST00000546983.1_3, *NKX2‐1_005*) containing different 5′‐untranslated region (5′‐UTR) first exons. Expression profile according to Genotype‐Tissue Expression (GTEx) database (release V6p: dpGap Accession phs000424.v6.p1) showed that all isoforms except *NKX2‐1_003* were highly expressed in the normal lung and thyroid gland, suggesting site‐specific expression. The transcripts *NKX2‐1_001*, *NKX2‐1_002*, and *NKX2‐1_004* contained homeodomains, while *NKX2‐1_005* did not. With respect to expression level, *NKX2‐1_001* had the highest expression, followed by *NKX2‐1_002*, whose expression level was nearly equal to that of *NKX2‐1_004* (Figure 1A). Further computational analysis using FANTOM5 data revealed 17 TSSs in the human genome. Among them, 14 TSS regions (82%), including the TSSs 1–12, 13, and 15, were detected in LADs examined in this study (Figure 1B and supplementary material, Table S2). Thus, CAGE assay combined with full‐length RNA‐seq demonstrated that all transcript isoforms except *NKX2‐1_003* were expressed in LADs. *NKX2‐1_001* was regulated by TSSs 7 and 13, whereas *NKX2‐1_002* was regulated by TSSs 1, 3, 5, 8, 12, and 15. Furthermore, six TSSs (TSSs 2, 4, 6, 9, 10, and 11) regulated *NKX2‐1_004* and *NKX2‐1_005*, containing exon 1 (Figure 1C). Expression levels of TSSs that regulated the same 5′‐UTR first exons were correlated with each other (Figure 1D).

### TSS‐level expression of *NKX2‐1/TTF‐1* was associated with LAD prognosis

**Figure 2:**
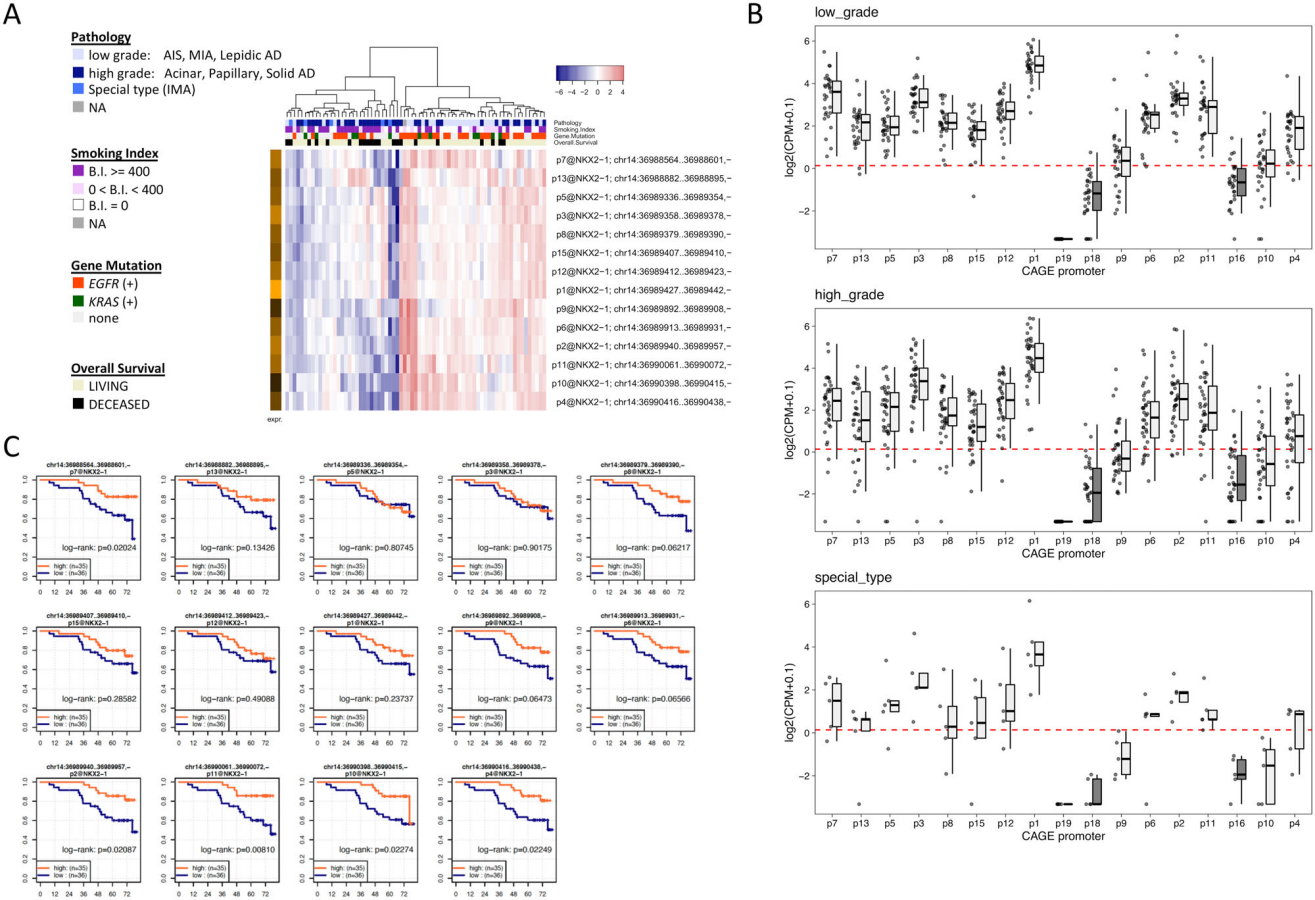
TSS expression level of TTF‐1 (*NKX2‐1/TTF‐1)* in LAD. (A) Seventy‐one LADs are clustered based on enrichment of the expression of 14 *NKX2‐1/TTF‐1* TSSs. (B) Box plots of the TSS regions of *NKX2‐1/TTF‐1* in each histological grade. Expression was quantified as CPM. Each box indicates the mean and upper and lower quartiles, whereas the bar indicates the range. Both low‐ and high‐grade adenocarcinoma as well as special type (IMA) expressed 14 TSSs. (C) Comparison of OS in tumours with each TSS expression level. Note that the expression level of TSS region 11 is the most associated with prognosis in patients with LAD (log‐rank *p* = 0.00810).

In the discovery data set, 71 LADs were clustered based on enrichment of the expression of isoforms from 14 TSSs of *NKX2‐1/TTF‐1*. A subset of isoforms from TSSs was highly expressed or decreased in each case (Figure 2A). Furthermore, conventional LAD as well as IMA, LAD of special type, used all 14 of the TSSs (Figure 2B). With respect to the malignant potential of TSSs, LADs with low expression of isoforms from TSS region 11, which regulated exon 1 in *NKX2‐1/TTF‐1*, were the most significantly associated with poor prognosis (*p* = 0.00810). Furthermore, low expression of isoforms from other TSSs that regulate exon 1, including TSSs 2 (*p* = 0.02087), 4 (*p* = 0.02249), and 10 (*p* = 0.02274) was significantly associated with poor prognosis. In contrast, expression of isoforms from other TSSs, including those regulating *NKX2‐1_001* and *NKX2‐1_002* was not significantly correlated with prognosis, with the exception of TSS region 7 (*p* = 0.02024) (Figure 2C).

### Detection of *NKX2‐1/TTF‐1* exon 1 in FFPE LAD tumour tissues

**Figure 3:**
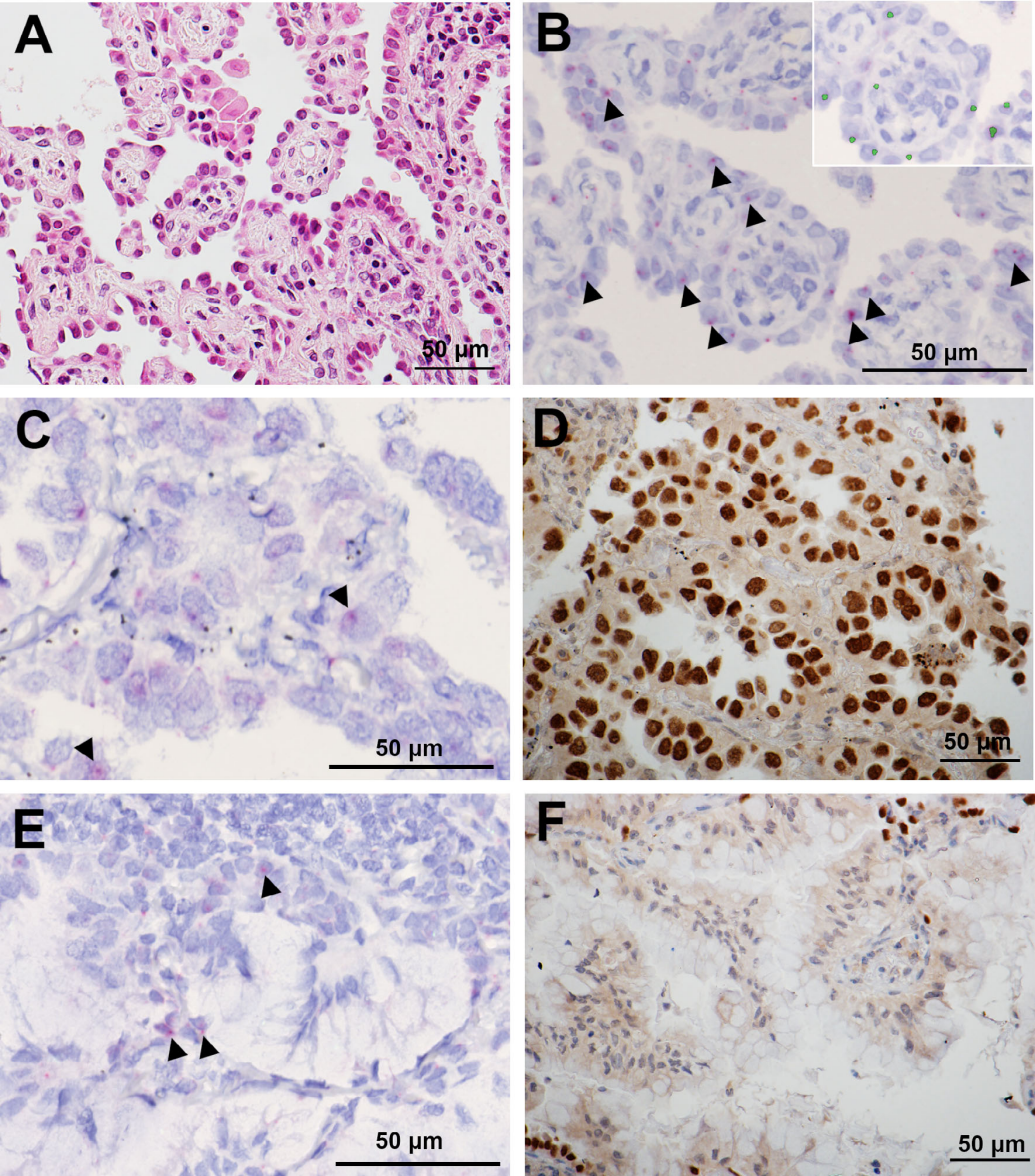
Detection of TTF‐1 (*NKX2‐1/TTF‐1*) exon 1 in FFPE tissues using RNAscope. Representative (A) H&E staining of a lung adenocarcinoma and (B) RNAscope in FFPE tumour tissue. The designed probe binds to the region of *NKX2‐1* exon 1 and is visualised as red dots. inForm Advanced Image Analysis recognises red dots (arrowheads) for quantitative image analysis. Recognised dots are visualised as green (inset). (C) Representative RNAscope image of a lung adenocarcinoma expressing *NKX2‐1/TTF‐1* exon 1 (arrowheads), which (D) exhibits TTF‐1 protein expression. (E) Representative RNAscope image of an invasive mucinous adenocarcinoma expressing *NKX2‐1/TTF‐1* exon 1 (arrowheads), which (F) lacks TTF‐1 protein expression.

The clinicopathological impact of expression of *NKX2‐1/TTF‐1* exon 1 was further examined. Expression of *NKX2‐1/TTF‐1* exon 1 was evaluated using RNAscope and a designed probe targeting *NKX2‐1/TTF‐1* exon 1 in FFPE tissues. First, the association of expression of *NKX2‐1/TTF‐1* exon 1 was examined, which was detected by CAGE and RNAscope in the discovery data set, and whose tumours exhibited high‐ to intermediate‐grade histology (acinar, papillary, and solid adenocarcinoma), indicating that there were correlations among them (*p* = 0.0020) (see supplementary material, Figure S2). Next, RNAscope was performed in the validation data set consisting of 664 LADs. The median optical density for *NKX2‐1/TTF‐1* exon 1 in the 664 LADs in the validation data set was 314.8, and 602 (90.7%) tumours expressed *NKX2‐1/TTF‐1* exon 1 (Figure 3).

### LAD clinicopathological characteristics of *NKX2‐1/TTF‐1* exon 1 expression

**Figure 4:**
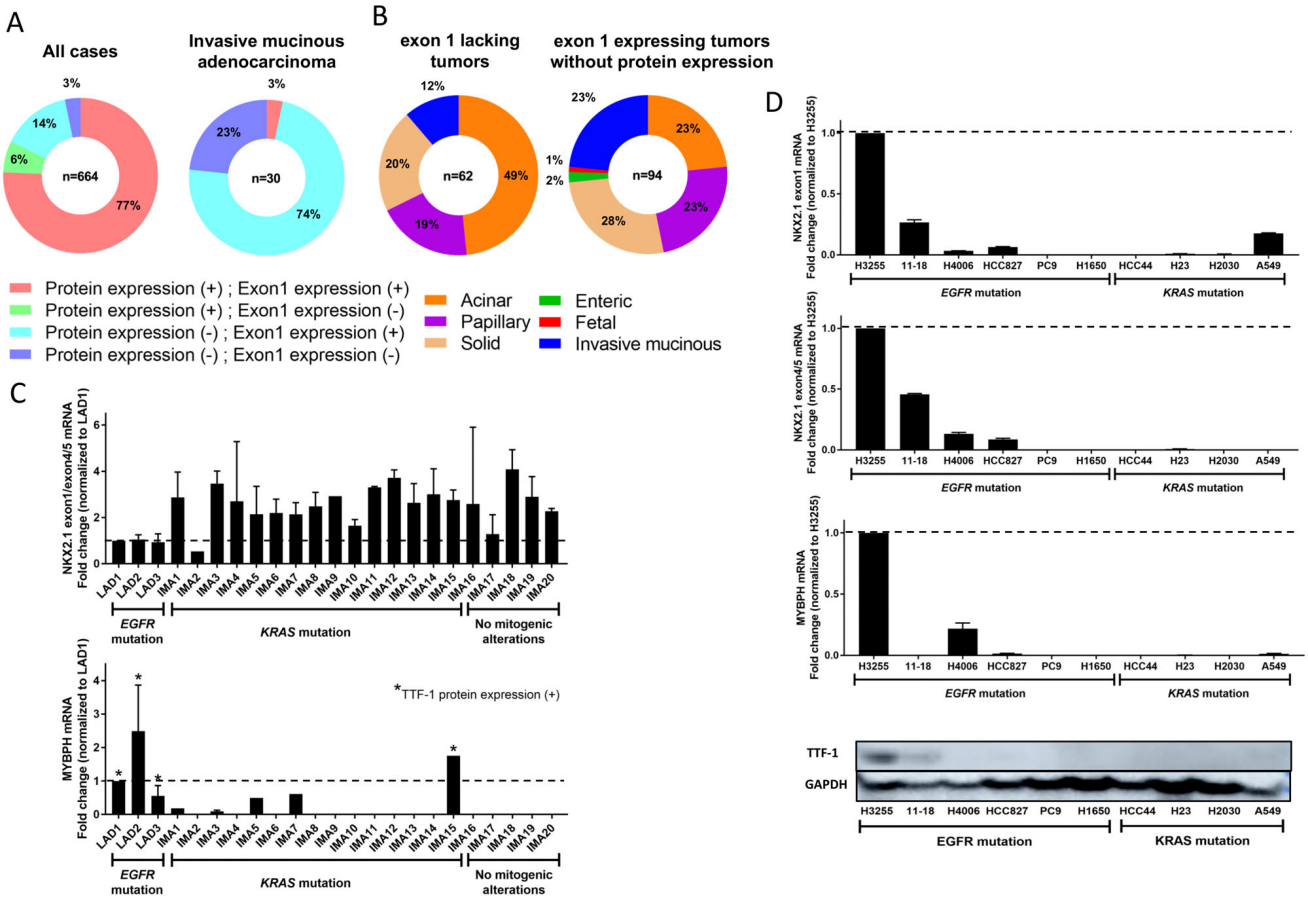
A subset of LAD exhibits TTF‐1 (*NKX2‐1/TTF‐1*) exon 1 mRNA expression, but lacks TTF‐1 protein expression. (A) Pie charts showing the fraction of LADs (left panel) and IMA (right panel) that harbour the indicated exon 1 and TTF‐1 protein expression. Red indicates tumours with expression of *NKX2‐1/TTF‐1* exon 1 and TTF‐1 protein expression. Green indicates tumours without expression of *NKX2‐1/TTF‐1* exon 1, but with TTF‐1 protein expression. Light blue indicates tumours with expression of *NKX2‐1/TTF‐1* exon 1, but without TTF‐1 protein expression. Blue indicates tumours without both expression of *NKX2‐1/TTF‐1* exon 1 and TTF‐1 protein expression. (B) Pie charts showing the fraction of histological subtypes that exhibit no expression of *NKX2‐1/TTF‐1* exon 1 (left panel) and expression of *NKX2‐1/TTF‐1* exon 1, but lack TTF‐1 protein expression (right panel). (C) Results of qPCR for 20 cases of IMA, as well as three *EGFR*‐mutated LADs, showing that 19 cases exhibited higher exon 1 expression relative to that of exon 4/5, compared to *EGFR‐*mutated adenocarcinoma that was immunohistochemically positive for TTF‐1 (upper panel). *MYBPH*, a direct transcriptional target of NKX2‐1/TTF‐1, was rarely expressed in IMA with the exception of IMA15 exhibiting TTF‐1 protein expression (lower panel). (D) Expression of *NKX2‐1/TTF‐1* exon 1 and exon 4/5, *MYBPH*, as well as TTF‐1 protein expression detected by western blots in a panel of 10 lung cancer cell lines. An *EGFR*‐mutated cell line (H3255) showing high expression of *NKX2‐1/TTF‐1* exon 4/5, and *MYBPH* also expresses TTF‐1 in western blots, in contrast to no or subtle TTF‐1 protein expression in other cell lines. Note that A549 cells exhibited exon 1 expression relative to the *EGFR*‐mutated cell line (H3255), despite lacking expression of *NKX2‐1/TTF‐1* exon 4/5 and TTF‐1 protein expression.

To clarify LAD characteristics with no *NKX2‐1/TTF‐1* exon 1 expression, the clinicopathological factors in 664 LADs were evaluated. The clinicopathological characteristics of 62 LADs with no expression of *NKX2‐1/TTF‐1* exon 1 are summarised in Table 2. LADs with no expression of *NKX2‐1/TTF‐1* exon 1 were significantly associated with older age (*p* = 0.0006), no TTF‐1 immunoreactivity (*p* = 0.0009), and *EGFR* wild‐type tumours (*p* = 0.0009). However, there was no significant correlation between expression of *NKX2‐1/TTF‐1* exon 1 and LAD histological features, such as micropapillary or cribriform patterns, that are unfavourable prognostic factors in LAD [24]. Furthermore, 49 (79%) and 51 (82%) LADs with no *NKX2‐1/TTF‐1* exon 1 expression exhibited an *EGFR* and *KRAS* wild‐type genotype, respectively. In contrast, 94 (14%) LADs exhibited *NKX2‐1/TTF‐1* exon 1 expression without TTF‐1 protein expression. Of those, solid adenocarcinoma was the most frequent histological subtype (27%), followed by acinar, papillary adenocarcinoma, and IMA (23%). Interestingly, IMA frequently (73%) exhibited the same expression pattern, and other LADs of a special type, including one fetal adenocarcinoma and two enteric adenocarcinomas, also exhibited a similar expression pattern (Figure 4A). Whether IMA exhibited *NKX2‐1/TTF‐1* exon 1 expression was further validated using qPCR, revealing that 19 of 20 (95%) cases exhibited higher exon 1 expression relative to that of exon 4/5, compared to *EGFR‐*mutated adenocarcinoma that was immunohistochemically positive for TTF‐1 (Figure 4B).

**Table 2:** Clinicopathological characteristics of LAD with *NKX2‐1/TTF‐1* exon 1 expression.

	Tumour without exon 1 expression (*n* = 62)	Tumour with exon 1 expression (*n* = 602)	Correlation (*p*)
Median age (range)	71.66 (41–85)	67.38 (28–89)	0.0006
Gender			0.0610
Female	22	292	
Male	40	310	
Smoking index			0.2827
≤100	22	275	
101–400	7	63	
≥401	33	262	
Unknown	0	2	
Size (mm)			0.4728
≤20	17	219	
21–30	20	180	
31–50	16	142	
≥51	9	61	
Nodal status			0.2544
N0	40	430	
N1/N2/N3	22	172	
TNM stage			0.2967
0–I	34	371	
II–IV	28	231	
Histological subtype			0.0618
Acinar adenocarcinoma	30	275	
Papillary adenocarcinoma	12	194	
Solid adenocarcinoma	13	98	
Micropapillary adenocarcinoma	0	7	
IMA	7	23	
Enteric adenocarcinoma	0	3	
Fetal adenocarcinoma	0	2	
Histological grade			0.0259
Intermediate/high grade	55	574	
Special	7	28	
Lymphovascular invasion			0.2425
Absent	40	342	
Present	22	260	
Pleural invasion			0.1648
Absent	34	384	
Present	28	218	
Micropapillary pattern			0.3173
Absent	56	516	
Present	6	86	
Cribriform pattern			0.0871
Absent	52	546	
Present	10	56	
Clear cell features			0.1570
Absent	48	508	
Present	14	94	
Signet ring cell features			0.8539
Absent	61	594	
Present	1	8	
TTF‐1 immunoreactivity			0.0009
Absent	20	94	
Present	42	508	
*EGFR* mutation			0.0009
Absent	49	345	
Present	13	257	
*KRAS* mutation			0.5600
Absent	51	512	
Present	11	90	

### A549 cells expressed *NKX2‐1/TTF‐1* exon 1

To validate the disproportionate expression levels between *NKX2‐1/TTF‐1* exon 1 and exon 4/5 observed in FFPE tumour tissues, expression of both *NKX2‐1/TTF‐1* exon 1 and exon 4/5 was evaluated in a panel of 10 lung cancer cell lines, consisting of six *EGFR‐*mutated and four *KRAS‐*mutated cell lines. H3255 expressed the highest level of *NKX2‐1/TTF‐1* exon 4/5 mRNA, which is consistent with a previous study [26]. Overall, the expression levels of NKX2‐1/TTF‐1 exon 1 were positively correlated with those of exon 4/5. However, A549, a *KRAS‐*mutated cell line, expressed *NKX2‐1/TTF‐1* exon 1, despite lacking the expression of *NKX2‐1/TTF‐1* exon 4/5, MYBPH, and TTF‐1 protein expression (Figure 4C).

### Clinical outcomes

**Figure 5:**
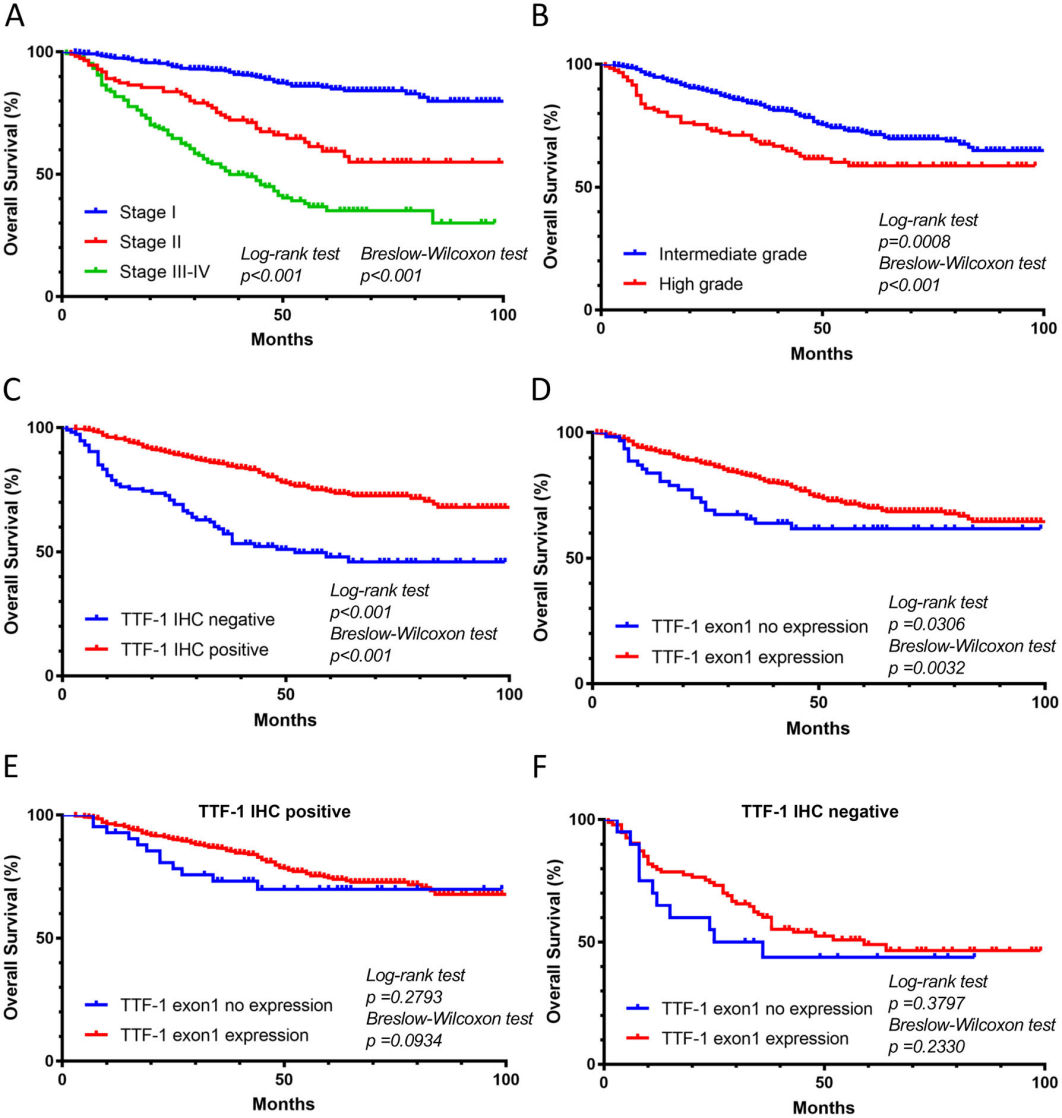
Kaplan–Meier curves of OS of 664 patients with LAD after surgical resection. Comparison of OS according to (A) stage, (B) histological grade, (C) tumour TTF‐1 protein expression, and (D) tumour *NKX2‐1/TTF‐1* exon 1 expression. (E and F) Comparison of OS stratified by *NKX2‐1/TTF‐1* exon 1 expression in patients with immunohistochemically TTF‐1‐positive (E) or ‐negative (F) tumours.

The median follow‐up period after surgery for all patients in the validation data set was 53.2 months. Overall survival (OS) rate was significantly associated with pathological stage (log‐rank test, *p* < 0.0001; Breslow–Wilcoxon test, *p* < 0.0001) and pathological grade (log‐rank test, *p* = 0.0008; Breslow–Wilcoxon test, *p* < 0.0001) (Figure 5A,B). Patients with TTF‐1‐positive tumours had significantly favourable OS (log‐rank test, *p* < 0.0001; Breslow–Wilcoxon test, *p* < 0.0001) (Figure 5C), which is consistent with previous reports [27]. In addition, patients whose tumour exhibited no expression of *NKX2‐1/TTF‐1* exon 1 had significantly shorter median OS (log‐rank test, *p* = 0.0306; Breslow–Wilcoxon test, *p* = 0.0032); however, these differences were barely significant compared to TTF‐1 protein expression (Figure 5D). Furthermore, among tumours with TTF‐1 protein expression, median OS was shorter in those patients whose tumours exhibited no expression of *NKX2‐1/TTF‐1* exon 1 than in those patients with expression of *NKX2‐1/TTF‐1* exon 1, although these differences were not significantly different; similar results were obtained among TTF‐1‐negative LADs, suggesting that a tumour suppressive role of *NKX2‐1/TTF‐1* transcript isoforms lacking exon 1 may be independent of TTF‐1 protein expression (Figure 5E,F). In subgroup analyses of *EGFR‐* and *KRAS‐*mutated cases, there was no significant difference in survival between tumours with and without expression of *NKX2‐1/TTF‐1* exon 1, although the latter had shorter OS (41 months in *EGFR‐*mutated cases and 36 months in *KRAS‐*mutated cases) (see supplementary material, Figure S3).

## Discussion

To the best of our knowledge, this is the first study to assess the association between *NKX2‐1/TTF‐1* isoforms and various clinicopathological parameters in LAD. These results of TSS‐level expression of *NKX2‐1/TTF‐1* revealed that LAD cells use at least 80% of NKX2‐1 TSSs, suggesting that each TSS and transcript isoform could play a distinct role in lung tumourigenesis, which contributes to the degree of heterogeneity of tumours. Furthermore, it was demonstrated that LAD with low expression of isoforms from TSS region 11, as well as from TSSs 2, 4, and 10, which regulate *NKX2‐1_004* and *NKX2‐1_005* containing exon 1, exhibited poor prognosis. Among *NKX2‐1/TTF‐1* isoforms, *NKX2‐1_001* and *NKX2‐1_002* have been the focus of intense research activities. ‘The proximal major promoter’ that regulates *NKX2‐1_001* contains a TATA‐like element and binding site for Forkhead box A1 (FOXA1) (*HNF3α*), FOXA2 (*HNF3β*), and GATA‐binding protein 6 (*GATA6*), all of which are known to be crucially involved in lung development, whereas ‘the minor distal promoter’ that regulates *NKX2‐1_002* is modulated by the transcription factors SP1 and SP3 [28]. However, the role of *NKX2‐1_004* and *NKX2‐1_005* containing exon 1 in lung development, homeostasis, and tumourigenesis remains largely unclear. Interestingly, *NKX2‐1/TTF‐1* exon 1 is conserved across multiple species, including non‐primate species, except fish, in contrast to other exons in *NKX2‐1/TTF‐1* that are highly to completely conserved, regardless of species, on the University of California Santa Cruz Genome Browser [29], suggesting that *NKX2‐1_004* and *NKX2‐1_005*, especially *NKX2‐1_004* containing a homeodomain, may play a pivotal role in the development and regulation of homeostasis of peripheral lung epithelial cells.

In the present study, LADs with no expression of *NKX2‐1/TTF‐1* exon 1 were associated with poor survival outcomes. As *NKX2‐1_005* lacks a homeodomain that binds DNA in a sequence‐specific manner and transcriptionally activates target genes, reduced expression of *NKX2‐1_004* rather than *NKX2‐1_005* may induce aggressiveness in LAD, suggesting a tumour suppressive role of *NKX2‐1_004*. Likewise, previous reports [27] along with these data show that reduced expression of TTF‐1 is significantly associated with unfavourable prognosis in patients with LAD, indicating a tumour suppressive function of NKX2‐1/TTF‐1 in lung tumourigenesis. However, loss‐of‐function and gain‐of‐function studies in human lung carcinoma and transformed cells support a role of *NKX2‐1* as an oncogene [7, 30, 31, 32, 33]. Furthermore, haploinsufficiency or conditional knockout of Nkx2‐1/Ttf‐1 in a transgenic mouse model leads to enhanced development of *Kras*‐mutated lung tumours, in contrast to suppression of *Egfr‐*mutated lung tumours [26]. Notably, MYBPH, a direct transcriptional target of NKX2‐1/TTF‐1, reduces cell motility and metastasis in *KRAS‐*mutated cell lines [34]. In addition, *NKX2‐1/TTF‐1*‐regulated microRNA‐532‐5p has a tumour suppressive role by targeting *KRAS* in LADs [35]. These data suggested that *NKX2‐1/TTF‐1* had both an oncogenic and suppressive role in lung tumourigenesis, which could be dependent on mitogenic driver mutations. Although the specific function of *NKX2‐1_004* in both LAD and normal lung tissues remains unclear at present, recent comprehensive epigenome and transcriptome analyses using Tracing Enhancer Networks using Epigenetic Traits (TENET) reveals that NKX2‐1 is the top transcriptional regulator inactivated in LAD, and is linked to over a hundred inactivated enhancers [36]. Further studies by other approaches will be needed to elucidate transcriptional regulation of *NKX2‐1* in lung cancer development and the role of each splice variant in different genetic backgrounds. Nevertheless, it was demonstrated that no expression of *NKX2‐1/TTF‐1* exon 1 was frequently detected in *EGFR* and *KRAS* wild‐type tumours, suggesting that a tumour suppressive role of *NKX2‐1_004* might be independent of such oncogenic alterations.

IMA is a unique histological variant of LAD, which commonly lacks TTF‐1 expression and expresses hepatocyte nuclear factors (HNFs), including HNF4α [25]. An inactivating mutation or epigenetic silencing of *NKX2‐1/TTF‐1* downregulates its protein expression. Recently, while *NKX2‐1/TTF‐1* inactivation mutations are rare, they are found in TTF‐1‐negative LADs, especially in IMA (33–43%) [37, 38]. Furthermore, *NKX2‐1/TTF‐1* is hypermethylated in the remaining TTF‐1‐negative cases; however, neither inactivation mutations nor hypermethylation is detected in some TTF‐1‐negative LADs [37], suggesting that other mechanisms of epigenetic silencing, such as microRNA and histone modification, may be involved in the downregulation of NKX2‐1/TTF‐1. In the present study, A549 cells exhibited expression of *NKX2‐1/TTF‐1* exon 1, despite lack of expression of *NKX2‐1/TTF‐1* exon 4/5 and subsequent TTF‐1 protein expression *in vitro*, which is consistent with a previous study showing that neither *NKX2‐1_001* nor *NKX2‐1_ 002* transcripts are detected in A549 cells [14]. Moreover, it was identified that most IMA exhibited the same expression pattern. Thus, it is possible that a subset of LADs, including IMA, use an alternative TSS and subsequently express *NKX2‐1_005* lacking a homeodomain. It still unclear whether *NKX2‐1_005* translates into protein. However, accumulated evidence shows that long non‐coding RNAs play a pivotal role in gene regulation [39]. RNAs insufficiently spliced from *NKX2‐1_005* are retained in the nucleus and might be linked with their specific subcellular localisations and functions in IMA, and may distinguish the biological behaviour of IMA from that of other conventional LADs. Alternatively, the protein translated from *NKX2‐1_005* might act in a dominant‐negative manner, affecting interactions of other isoforms with cofactors, and thus affecting TTF‐1 binding to its cognate sites.

However, this study has some limitations including undetermined transcriptome profiles of *NKX2‐1* in lymph nodes or in distant metastases, as NKX2‐1/TTF‐1 shows different functions depending on cell conditions [7]. Additional studies are required to clarify the clinicopathological impact of *NKX2‐1/TTF‐1* exon 1 expression in LAD using samples from metastatic sites or recurrent disease.

In summary, these transcriptome and clinicopathological analyses reveal that LADs harbour at least 14 TSSs of *NKX2‐1/TTF‐1*, and decreased expression of *NKX2‐1/TTF‐1* transcript isoforms with exon 1, such as *NKX2‐1_004*, lead to poor prognosis in patients with LAD, most of which had a *EGFR/KRAS* wild‐type genotype. *In situ* hybridisation for Epstein–Barr virus‐encoded RNA is practically used in the pathology laboratory. Likewise, detection of specific exons or exon junctions by *in situ* hybridisation may be useful to further classify LADs. While these results are valuable as an indicator of a patient's prognosis, further investigation targeting cancer‐specific splice variants, such as *NKX2‐1_005* in IMA, may be novel potential targets for LAD.

## Author contributions statement

KSa, TH, SKi, MK, TY and TS provided pathological information. KT and KSu provided patient's clinical information. KSa and SS carried out RNAscope assay and image analysis. MH, KT and SKo carried out bioinformatics analysis of the CAGE data set. KSa, TH, YS, KK, MI and TS conceived experiments and analysed data. All authors were involved in writing the paper and had final approval of the submitted and published versions. TH takes full responsibility for the work as a whole, including the study design, access to data, and the decision to submit and publish the manuscript.

## Supporting information


**Figure S1.** Correlation of optical density and survival
**Figure S2.** Correlation analysis between CAGE and RNAscope
**Figure S3.** Kaplan–Meier curves of OS of 664 patients with LAD after surgical resection

**Table S1.** RNAscope probe design
**Table S2.** Expression level of NKX2‐1/TTF‐1 promoters in 71 LADs
