# Supplementary material for: Transcription start site‐level expression of thyroid transcription factor 1 isoforms in lung adenocarcinoma and its clinicopathological significance
Source: J Pathol Clin Res. 2021 May 20;7(4):361–74. doi: 10.1002/cjp2.213 (PMC8185369; doi:10.1002/cjp2.213)
Supplement: Supplementary file 1 — Figure S1. Correlation of optical density and survival Figure S2. Correlation analysis between CAGE and RNAscope Figure S3. Kaplan–Meier curves of OS of 664 patients with LAD after surgical resection [file CJP2-7-361-s001.docx]

**Transcription start site-level expression of thyroid transcription factor 1 isoforms in lung adenocarcinoma and its clinicopathological significance**

K Sano *et al*. *J Pathol Clin Res* DOI: 10.1002/cjp2.213

**Supplementary Figures**

**Figure S1.** Correlation of optical density and survival.

**Figure S2.** Correlation analysis between Cap Analysis of Gene Expression (CAGE) and RNAscope.

**Figure S3.** Kaplan-Meier curves of overall survival (OS) of 664 patients with lung adenocarcinoma after surgical resection .

**
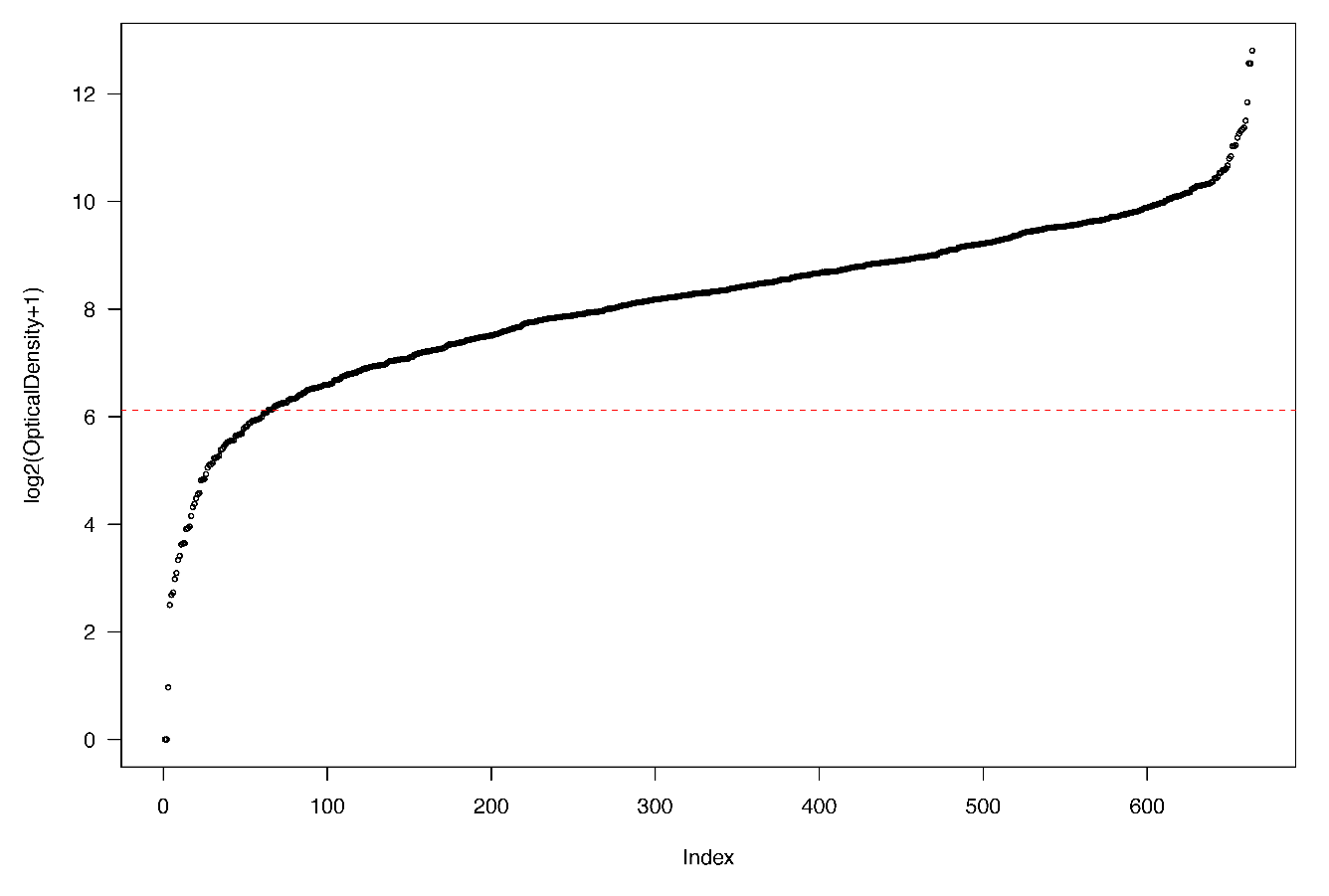
**

**Figure S1. Correlation of optical density and survival.** The black curve indicates the optical density distribution of 664 samples examined in this study. Red line indicated the cut-off value for optical density.

**
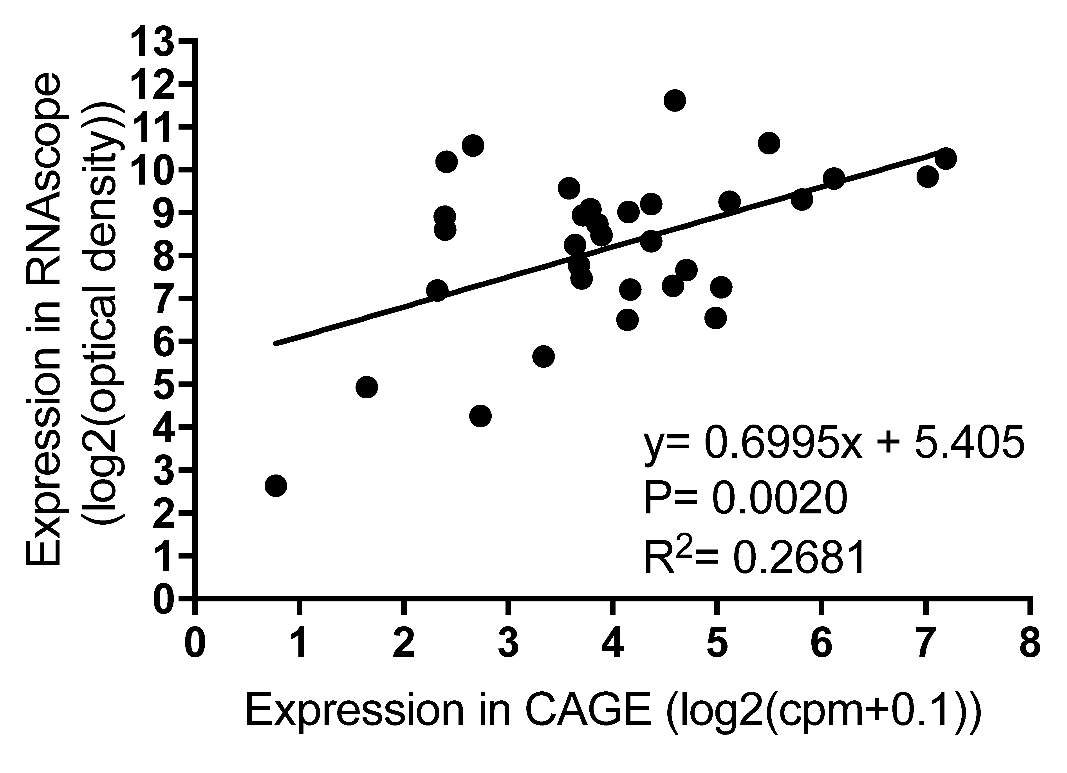
**

**Figure S2. Correlation analysis between Cap Analysis of Gene Expression (CAGE) and** **RNAscope.** In 33 LADs, expression of *NKX2-1/TTF1* exon 1, detected by CAGE, is significantly positively correlated with that of RNAscope (*p* = 0.0020).

**
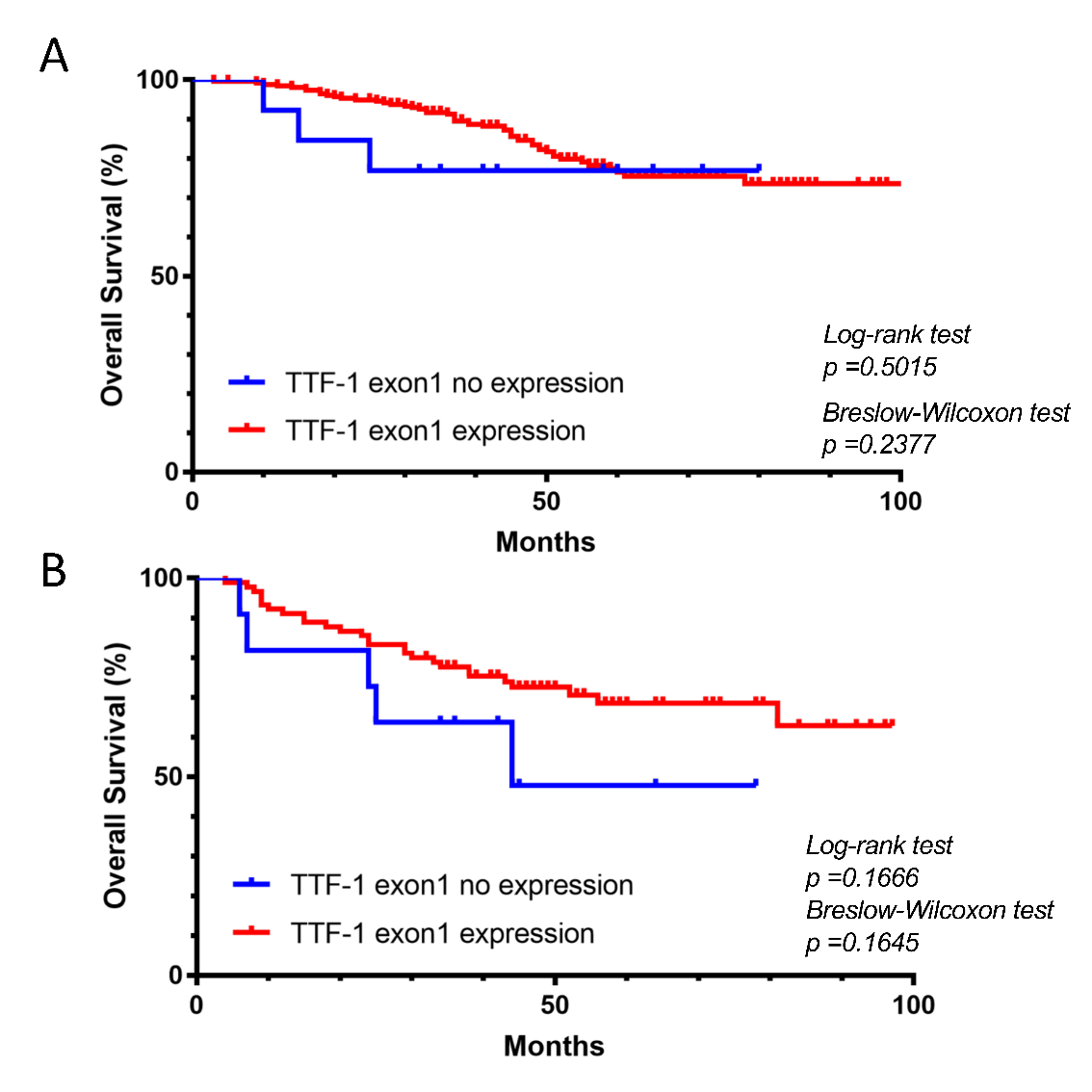
**

**Figure S3. Kaplan-Meier curves of overall survival (OS) of 664 patients with lung adenocarcinoma after surgical resection.** Kaplan-Meier estimation of OS. (A) Comparison of OS in patients with *EGFR*-mutated tumours that express *NKX2-1/TTF1* exon 1. (B) Comparison of OS in patients with *KRAS-*mutated tumours that express *NKX2-1/TTF1* exon 1.
